# Development, Testing, Parameterisation and Calibration of a Human PBPK Model for the Plasticiser, Di-(2-propylheptyl) Phthalate (DPHP) Using in Silico, *in vitro* and Human Biomonitoring Data

**DOI:** 10.3389/fphar.2021.692442

**Published:** 2021-09-02

**Authors:** Kevin McNally, Craig Sams, Alex Hogg, Annie Lumen, George Loizou

**Affiliations:** ^1^ Health and Safety Executive, Buxton, United Kingdom; ^2^ National Center for Toxicological Research, US Food and Drug Administration, Jefferson, AR, United States

**Keywords:** plasticiser, DPHP, PBPK, in silico, *in vitro*, biomonitoring, bayesian, markov chain Monte Carlo

## Abstract

A physiologically based pharmacokinetic model for Di-(2-propylheptyl) phthalate (DPHP) was developed to interpret the biokinetics in humans after single oral doses. The model was parameterized with *in vitro* and in silico derived parameters and uncertainty and sensitivity analysis was used during the model development process to assess structure, biological plausibility and behaviour prior to simulation and analysis of human biological monitoring data. To provide possible explanations for some of the counter-intuitive behaviour of the biological monitoring data the model included a simple lymphatic uptake process for DPHP and enterohepatic recirculation (EHR) for DPHP and the mono ester metabolite mono-(2-propylheptyl) phthalate (MPHP). The model was used to simultaneously simulate the concentration-time profiles of blood DPHP, MPHP and the urinary excretion of two metabolites, mono-(2-propyl-6-hydroxyheptyl) phthalate (OH-MPHP) and mono-(2-propyl-6-carboxyhexyl) phthalate (cx-MPHP). The availability of blood and urine measurements permitted a more robust qualitative and quantitative investigation of the importance of EHR and lymphatic uptake. Satisfactory prediction of blood DPHP and urinary metabolites was obtained whereas blood MPHP was less satisfactory. However, the delayed peak of DPHP concentration relative to MPHP in blood and second order metabolites in urine could be explained as a result of three processes: 1) DPHP entering the systemic circulation from the lymph, 2) rapid and very high protein binding and 3) the efficiency of the liver in removing DPHP absorbed via the hepatic route. The use of sensitivity analysis is considered important in the evaluation of uncertainty around *in vitro* and in silico derived parameters. By quantifying their impact on model output sufficient confidence in the use of a model should be afforded. This approach could expand the use of PBPK models since parameterization with in silico techniques allows for rapid model development. This in turn could assist in reducing the use of animals in toxicological evaluations by enhancing the utility of “read across” techniques.

## Introduction

Plastics have many useful applications due to their plasticity, which is the ability to be shaped and moulded. Plasticisers are different classes of chemicals used in the manufacture of plastics to create products of varying flexibilities and brittleness. Phthalates, which are some of the most commonly used plasticisers, are dialkyl- or alkylarylesters of 1, 2-benzenedicarboxylic acid. The length of the ester chain determines the industrial application, with alkyl chain lengths from three to 13 carbons widely used in polymers such as polyvinyl chloride (PVC).

Di-(2-propylheptyl) phthalate (DPHP), CAS No. 53306-54-0, marketed under the trade name Palatinol^®^10-P, is a high molecular weight branched phthalate ester which is used in the manufacture of polyvinyl chloride (PVC) products. DPHP can be found in cables, car interiors, carpet backing, pool liners, roofing membranes or tarpaulins, and consumer products such as shoes and artificial leather (Klein et al., 2018). Typical contents of DPHP in end-use products vary between 30 and 60% (w/w), 10.1–48.2% (w/w). While DPHP is a plasticizer predominantly recommended for technical applications, and has in the past been found in toys, food packaging and medicinal products (Klein et al., 2018) the European Union has advised against its use as well as not providing clearance for use in food contact materials^1^. DPHP, in common with other plasticizers, is not chemically bound in PVC products so can be released into the environment. Several studies have demonstrated the presence of DPHP in the general population (Wittassek and Angerer, 2008; Wittassek et al., 2011; Schutze et al., 2015; Schwedler et al., 2019; Schmidtkunz et al., 2019). However, when compared to human biomonitoring (HBM) health-based guidance values, (Schwedler et al, 2019), report no exceedance of the HBM-I^2^ value of 1 mg/L for DPHP (Sum of OH-MPHP + oxo-MPHP) (Apel et al., 2017).

Currently, there are no data on the toxicology of DPHP in humans and in contrast with other phthalates, studies in rats suggest that it is neither a reproductive toxicant nor an endocrine disruptor (BASF, 1995a; BASF, 2003; BASF, 2009; Furr et al., 2014). In other studies, increased liver weights, thyroid, and pituitary effects were observed following oral administration (BASF, 1995b; Union Carbide, 1997; Union Carbide, 1998; BASF, 2009). An oral reference dose of 0.1 mg/kg body weight per day in humans was derived from a benchmark dose of 10 mg/kg body weight per day for thyroid hypertrophy/hyperplasia in adult male rats (Bhat et al., 2014). The adverse effects that were observed with other phthalates that were related to metabolism of the parent phthalate to the primary monoester (Oishi and Hiraga, 1980; Foster et al., 1981; Sjöberg et al., 1986) are not reported to occur with DPHP. Large species-specific burdens of the primary monoester of di-(2-ethylhexyl) phthalate (DEHP) in venous blood were observed (Rhodes et al., 1986; Kessler et al., 2004; Kurata et al., 2012). Therefore, species-specific burdens of primary monoesters of DPHP in rat and human were proposed as a basis for a risk estimation of DPHP (Klein et al., 2016). A study involving the biological monitoring (BM) of human volunteers following administration of a single oral dose of DPHP was conducted for this purpose (Klein et al., 2018).

BM is the repeated controlled measurement of a chemical, its metabolites, or biochemical markers in accessible media such as urine, blood and saliva, exhaled air and hair (Manno et al., 2010). As a method of exposure assessment BM is considered superior to personal air or dermal deposition measurements. This is because more accurate estimates of body burden can be made, since BM measurements are a composite measure of multiple routes of exposure (Cocker and Jones, 2017). Differences in individual behaviour such as, personal hygiene and work rate, in addition to inter-individual differences in physiology and metabolism can be captured in BM measurements (Cocker and Jones, 2017). Uncertainty in external exposure assessment due to inter- and intra-individual variability can also be reduced by using BM if the measured biomarker, either parent chemical or metabolite(s), is proportionately related to the ultimate toxic entity (Boogaard et al., 2011). The ability to estimate organ and tissue dose or “tissue dosimetry” from body burdens calculated using BM should further improve the correlation of exposure to health effects.

Tissue dosimetry can be estimated with the application of physiologically based pharmacokinetic (PBPK) modelling. PBPK modelling is a powerful means of simulating the factors that determine tissue dose within any biological organism and consequently, it’s correlation with health effects (Andersen, 1995; Clewell III and Andersen, 1996; Andersen, 2003; Barton et al., 2007; Clewell et al., 2008; Loizou and Hogg, 2011). The value of PBPK models is that they are tools for integrating *in vitro*, in silico and *in vivo* mechanistic, pharmacokinetic, and toxicological information. PBPK models encode an explicit mathematical description of important anatomical, physiological and biochemical determinants of chemical uptake, distribution, and elimination. Thus, PBPK modelling is increasingly being used in chemical risk assessment (RA) (Chiu et al., 2007; Loizou et al., 2008; WHO, 2010).

In this study we present a PBPK model developed to interpret the venous blood concentrations of DPHP and its primary monoester metabolite, mono-(2-propylheptyl) phthalate (MPHP), and the urinary excretion of the two direct metabolites of MPHP, mono-(2-propyl-6-hydroxyheptyl) phthalate (OH-MPHP) and mono-(2-propyl-6-carboxyhexyl) phthalate (cx-MPHP). We adapted the model structure for di-isononyl-cyclohexane-1, 2-dicarboxylate (Hexamoll^®^ DINCH) described previously (McNally et al., 2019) to include a simple lymphatic uptake process for DPHP andenterohepatic recirculation of DPHP and MPHP (Yang et al., 2015). The model was parameterized using *in vitro* and in silico methods. These were measured intrinsic hepatic clearance scaled from *in vitro* to *in vivo* and predicted octanol–water partition coefficient (Log P_ow_) values which, in turn, were used to predict parameters such as plasma unbound fraction and tissue:blood partition coefficients (PCs). Also, the sufficiency and relevance of PBPK model structure and the sensitivity of model output to *in vitro* and in silico derived model parameters was investigated using an approach based on global sensitivity analysis (GSA). The latter is part of the ongoing development of a good PBPK modelling practice (Barton et al., 2007; Loizou et al., 2008; Barton et al., 2009; WHO, 2010; Paini et al., 2017; Ellison et al., 2019; Fabian et al., 2019).

## Materials and Methods

### Experimental

#### Chemicals

Pooled human microsomes were purchased from Tebu-bio (Peterborough, United Kingdom). The microsomes were prepared from a pool of 50 liver samples; mixed gender (20 mg protein ml^−1^). DPHP and MPHP were provided by BASF SE. All chemicals used were of analytical grade or higher.

#### Analysis

Samples were analysed by liquid chromatography (Shimadzu Prominence) with tandem mass spectrometry detection (AB Sciex API 3200) using electrospray ionisation. Ion optics, temperatures and gas flows were optimised on our individual system. All analyses used a Synergi Hydro-RP column (150 × 2 mm; 4 µ; Phenomenex) in conjunction with a methanol: 20 mM ammonium acetate (0.1% acetic acid) gradient. Sample injection volume was 2 µl.

#### *In vitro* Incubations

**FIGURE 1:**
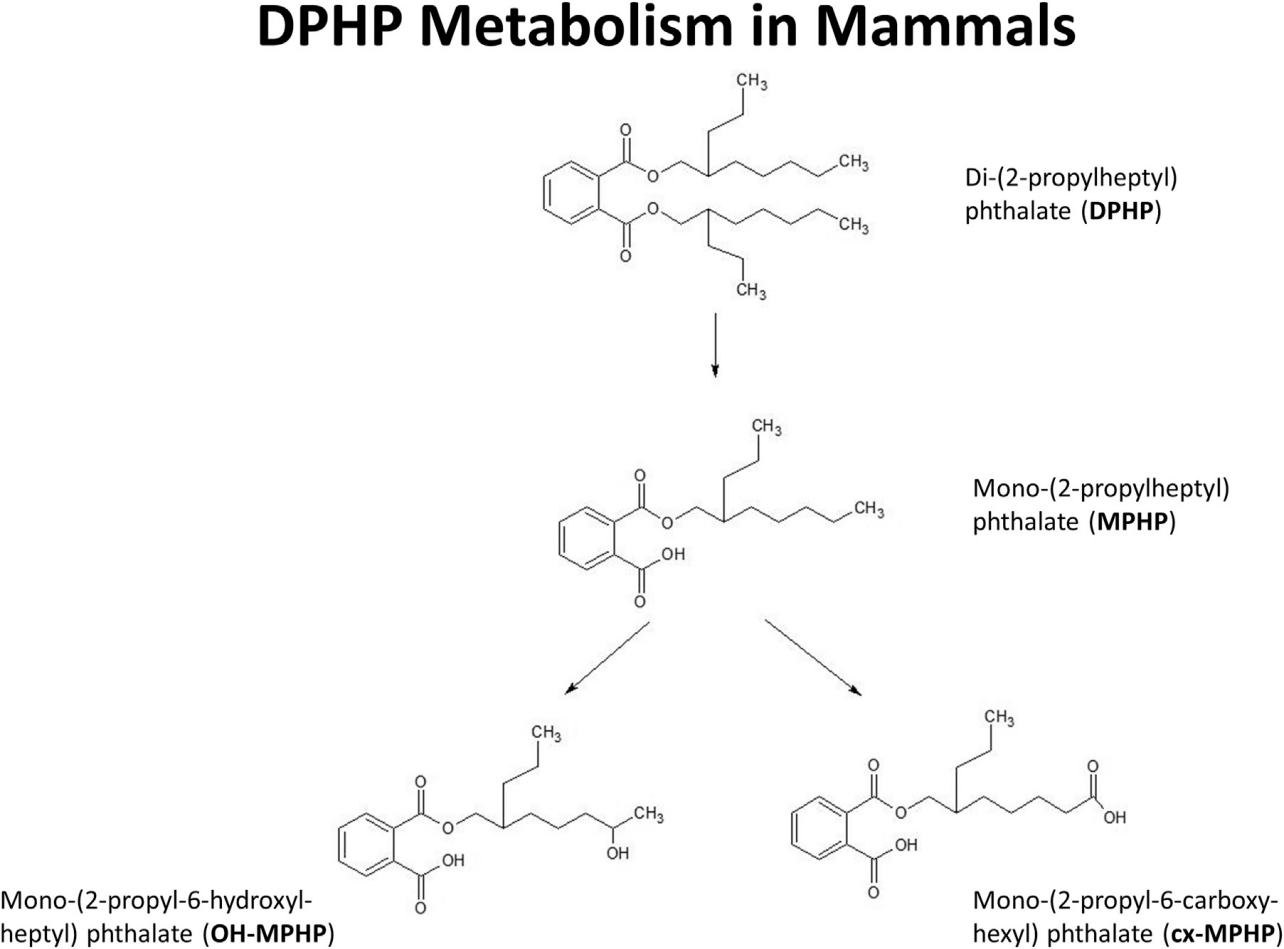
Postulated metabolism of DPHP in humans showing only those metabolites measured in human biological monitoring and described in the PBPK model.

The very high lipophilicity of DPHP resulted in the formation of an insoluble film on the surface of the reaction medium which precluded the measurement of *in vitro* clearance. Therefore, the measurement *in vitro* clearance of MPHP only was possible (Figure 1).

The NADPH regenerating system consisted of the following final concentrations: 1.3 mM NADP^+^; 3.3 mM glucose-6-phosphate; 5 mM magnesium chloride; 0.4 U/ml glucose-6-phosphate dehydrogenase; 50 mM phosphate buffer (pH 7.4). Final microsomal protein concentration was 0.5 mg/ml. Incubations were performed in polypropylene tubes and pre-warmed reaction mixtures were started by addition of substrate dissolved in acetonitrile. The final acetonitrile concentration was less than 1% and, typically, a substrate concentration of 10 µM was used (initial investigations were performed to check solubility in the reaction mixture). Incubations were conducted in a water bath at 37°C. At the time points chosen for measurement, tubes were mixed by inversion and an aliquot removed and quenched by adding to an equal volume of ice-cold methanol followed by centrifugation to precipitate the protein as a pellet. The supernatant was removed for analysis. Three replicates were sampled at each time point. Control incubations consisted of a reaction mix excluding glucose-6-phosphate dehydrogenase (for evaluation of non-specific binding) and reaction mix excluding microsomes (for evaluation of substrate stability).

The method of (Jones and Houston, 2004) was used to determine the *in vitro* half-life of substrate depletion. At least three independent incubations were performed and results were assessed visually for reproducibility. However, due to differences in sampling time points between experiments, results from individual incubations were not combined.

### Determination of *in vitro* Intrinsic Clearance

As described in (McNally, et al, 2019) *in vitro* intrinsic clearance for MPHP, *CL*_*in vitro*_ (ml min^−1^ mg^−1^ microsomal protein) in human hepatic microsomes was calculated using the half-life (*T*_*½*_) derived from the decay constant (*k*) using the following equations (Obach et al., 1997):

$$invitroT_{1/2}=\frac{\ln\left(2\right)}{k}$$ (1)

$$CL_{invitro=\frac{\ln\left(2\right)}{invitroT_{1/2}}\times \frac{mlincubation}{mgmicrosomes}}$$ (2)

Where, *ml incubation* is the volume (ml) of the incubation medium and *mg microsomes* is the mass (mg) of microsomes in the incubation medium.

### Calculation of *in vivo* Clearance

The intrinsic hepatic clearance CL_int_H_ (L h^−1^) was calculated using the following formula adapted from (Obach, 1999):

$$CL_{\mathrm{int}\_H}=CL_{invitro}\times MPY\times Vli\times 60$$ (3)

Where, *MPY* is the microsomal protein yield per g liver (mg g^−1^), *Vli* is mass of the liver (g) and the 60 converts from minutes to hours.

Whole liver plasma clearance *CL*_*H*_ (L h^−1^) was calculated assuming the well-stirred model of hepatic clearance taking into account the unbound fraction in plasma, *fu* and the red blood cells to plasma ratio, C_RBC_/C_P_, using the following equation (Yang et al., 2007):

$$CL_{H}=Q_{H}\cdot fu\cdot \frac{CL_{\mathrm{int}\_H}}{Q_{H}+fu\cdot CL_{\mathrm{int}\_H}/\left(C_{RBC}/C_{P}\right)}$$ (4)

Where, *Q*_*H*_ (L h^−1^) is the blood flow to the liver as a proportion of cardiac output.

The intrinsic gut clearance CL_int_gut_ was calculated similarly as described for hepatic clearance but substituting *MPY*_*gut*_ and *Vgu* for *MPY* and *Vli*, respectively, in Eq. 4. The resulting calculated CL_int_gut_ was used in place of CL_int_H_ for calculation of CL_gut_.

### Prediction of Log P_ow_ and Tissue:blood Partition Coefficients (PCs) and Plasma Fraction Unbound

The tissue:blood PCs and unbound fractions in plasma were calculated from the logarithm of the octanol–water partition coefficient, Log P_ow_ as described in McNally, et al. (2019). Briefly, the Log P_ow_ for DPHP and MPHP were calculated using the ACDLogP algorithm (Mannhold et al., 2009) implemented in the ACD/ChemSketch 2014 software (Table 1). The Log P_ow_s were input into two tissue-composition-based algorithms for the calculation of tissue:blood PCs. The method of (Poulin and Haddad, 2012), which was developed for the prediction of the tissue distribution of highly lipophilic compounds, defined as chemicals with a Log P_ow_ > 5.8, was used for DPHP (Table 1). The method of (Schmitt, 2008) , which was developed to predict the tissue distribution of chemicals with Log P_ow_ < 5.17, was used to predict the PCs of the monoester, MPHP (Table 1). The algorithm of (Poulin and Haddad, 2012) was implemented as a Microsoft^®^ Excel Add-in whereas a modified version of the algorithm of (Schmitt, 2008) was available within the httk: R Package for High-Throughput Toxicokinetics (Pearce et al., 2017). Where the tissue-composition-based algorithms did not provide a tissue:blood partition coefficient for a particular compartment, the value from a surrogate organ or tissue was assumed. These are presented in italicised text with the surrogate organ or tissue in brackets Table 1.

**TABLE 1:** Tissue:blood partition coefficients and plasma fraction unbound predicted using Log P_ow_.

	DPHP	MPHP
**Log Po:w**	10.83	5.3
**Tissue:blood partition coefficient**		
Adipose	63.4	29.10
Liver	5.89	54.8
Muscle	3.29	7.51
Blood cells	3.01	6.67
Gut	7.4	25.2
Spleen	3.7	12.20
Stomach^a^ (gut)	7.4	25.2
Rapidly Perfused (spleen)	3.7	12.20
Slowly Perfused (muscle)	3.29	7.51
**Plasma Fraction Unbound**	0.0025	0.0146

a Compartments in italics have surrogate values from another organ compartment. The corresponding surrogate organ compartment is in parentheses.

The fraction unbound (*fu*) was calculated from *log ((1-fu)/fu)* with the following equation:

$$fu=\frac{1}{10^{x}+1}$$ (5)

Where, x=0.4485logP-0.4782

When *x* is the equation for the prediction of *fu* for a chemical with a predominantly uncharged state at pH 7.4 (Lobell and Sivarajah, 2003) (Table 1).

### Calculation of Fraction Metabolised

The proportion of MPHP metabolised to cx- and OH-MPHP, represented by FracMetab (FracMetabcx to cx- MPHP and FracMetabOH to OH-MPHP) (Table 2) for each volunteer was estimated by expressing all the biological monitoring (BM) data (MPHP, OH-MPHP, cx-MPHP, oxo-MPHP) in moles and dividing the amount of cx- and OH-MPHP each by the sum total of all metabolites (Table 2).

**TABLE 2:** Volunteer specific parameters.

	Volunteers					
	A	B	C	D	E	F
Body weight (kg)	83	75	76	74	90	108
Dose (mg kg^−1^)	0.717	0.639	0.781	0.783	0.775	0.733
Fraction Metabolised						
FracMetab to cx_MPHP	0.02	0.017	0.02	0.023	0.018	0.018
FracMetab to OH_MPHP	0.396	0.340	0.374	0.359	0.334	0.329

### Biological Monitoring Data

The BM data described in (Klein et al, 2018) were kindly provided by Dr. Rainer Otter of BASF, SE. Briefly, DPHP was administered orally to six healthy male volunteers, aged between 30 and 64 years, weighing between 74 and 108 kg. A single dose of 738 ± 56 µg/kg BW DPHP was administered as an emulsion of 7% (w/v) in an aqueous saccharose solution (70% w/v) between 45 and 140 min after breakfast. The resulting respective doses for the six individuals were between 0.639 and 0.783 mg kg^−1^ body weight (Table 2).

### The PBPK Model

**FIGURE 2:**
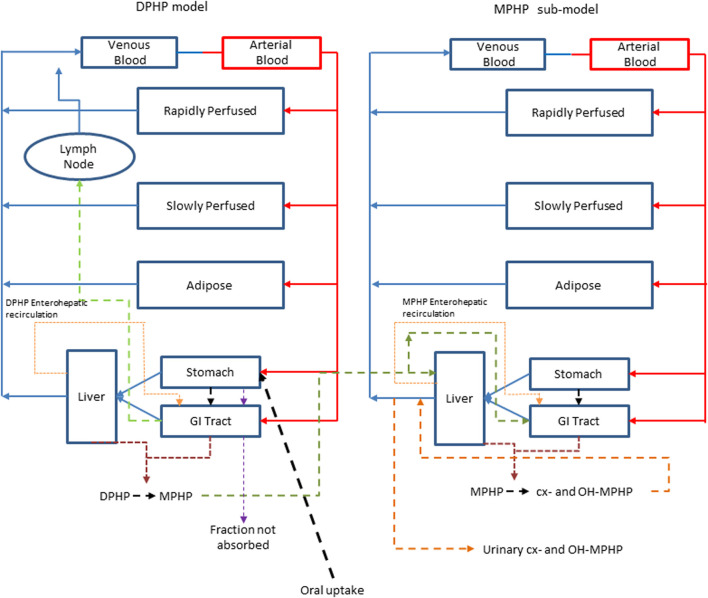
A schematic of the model for DPHP and sub-model for MPHP. The main model contained a lymphatic compartment which received a portion of oral dose from the stomach and GI tract. Urinary excretion of metabolites was ascribed to the sub-model.

A human PBPK model was developed to study the fate of DPHP following single oral doses. The initial model structure was based upon the PBPK model for the plasticizer DINCH described in (McNally, et al, 2019), with a minor modification to account for a large proportion of administered oral dose that is not absorbed, but eliminated by faecal excretion (Klein et al., 2018). The simulation of urinary excretion of metabolites as amount per hour was preferred as they were less variable than when expressed against creatinine to adjust for urinary dilution (Lermen et al., 2019). The model included a description of absorption from the stomach and gastro-intestinal (GI) tract and a simple model of the lymphatic system describing uptake of DPHP via the lacteals in the intestine and entering venous blood after bypassing the liver. Inclusion of a lymph compartment was based on the assumption that DPHP like di(2-ethylhexyl) phthalate (DEHP) binds like lipid to lipoproteins (Griffiths et al., 1988) which are formed in enterocytes and transported in the lymph of the thoracic duct (Kessler et al., 2012). The dose that entered the lymphatic system via the GI tract was coded as a fraction of the administered dose, with the complementary proportion entering the liver via the portal vein. The model described the metabolism of DPHP to MPHP in both liver and gut; therefore, both DPHP and MPHP entered systemic circulation via uptake from the gut. Enterohepatic circulation was also described as a possible explanation of the second small peak in urinary metabolite concentration observed in several datasets. A sub-model was added to describe the kinetics of MPHP, with the two models connected through the gut and liver compartments. The model for DPHP differed from the sub-model only with the presence of a lymphatic component and the MPHP sub-model describing urinary excretion of metabolites. Both models had a stomach and GI tract draining into the liver and systemically circulated to adipose, blood (plasma and red blood cell) and slowly and rapidly perfused compartments. First order elimination constants described the removal of second order metabolites OH-MPHP and cx-MPHP from blood into the urine. A representation of the model structure is given in Figure 2. The model code is available in Supplementary Materials.

**FIGURE 3:**
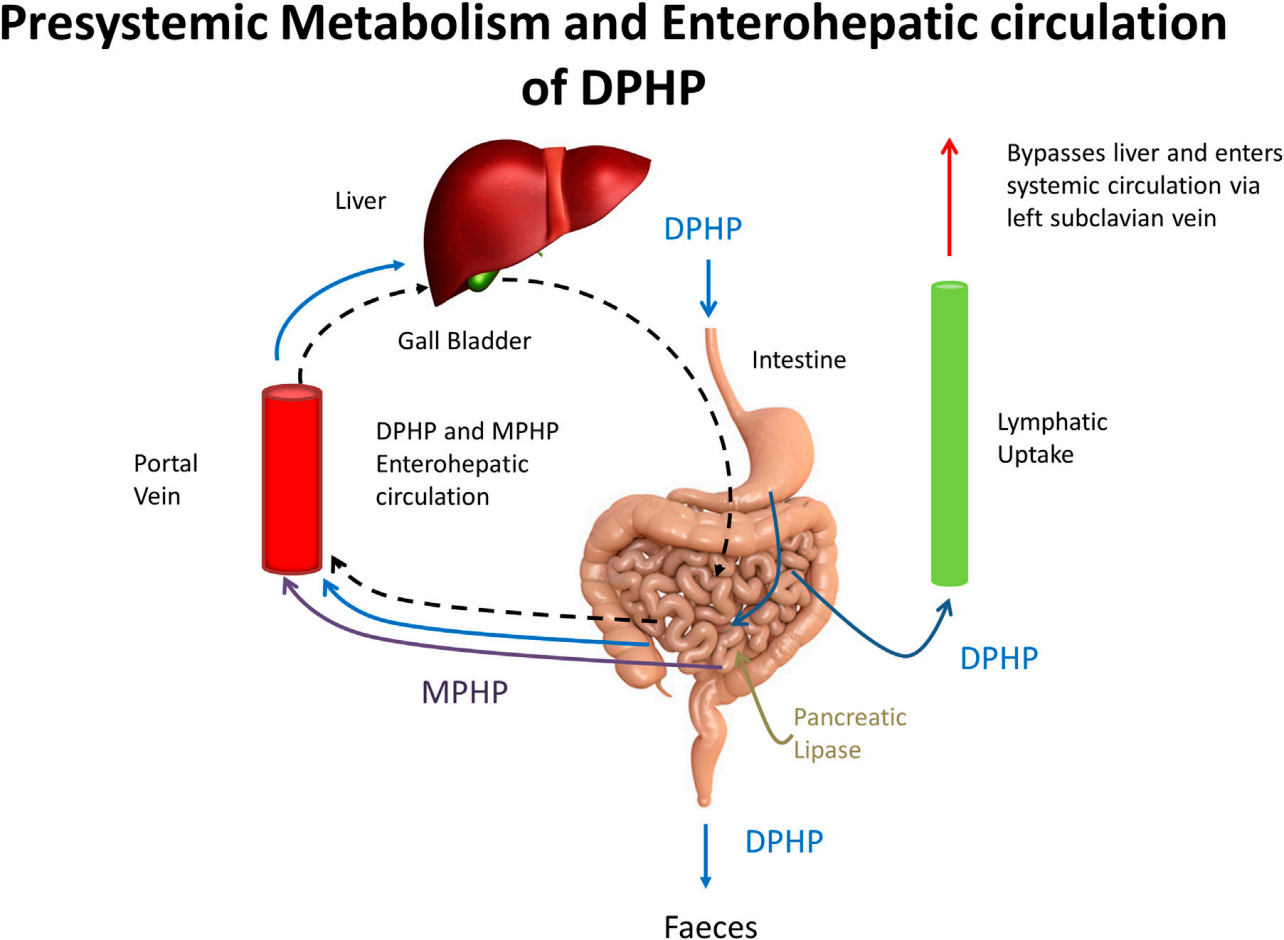
A schematic showing pre-systemic metabolism and enterohepatic recirculation, systemic and lymphatic uptake of DPHP and uptake of MPHP from gastrointestinal tract.

The baseline model was subsequently refined using an iterative model development process to better represent the trends in BM (blood and urine) data from the human volunteer study reported in (Klein et al., 2018). Techniques for uncertainty and sensitivity analysis (described in the statistical analysis section) were deployed, at each iteration of model development, to establish the bounding behaviour of the model and the key uncertain parameters that the model outputs under study were sensitive to. The improvements and a brief justification are described in the points below:

1. The model was adapted to account for a majority fraction of DPHP passing through the GI tract without being absorbed, as suggested by BM data, ranging from 75% (Wittassek and Angerer 2008; Leng et al., 2014) to 94% (Klein et al., 2018). *FracDOSEHep* and *FracDOSELymph* modelled the fractions of the administered dose entering hepatic and lymphatic circulation respectively, with the complementary fraction (1 – *FracDOSEHep – FracDOSELymph)* passing directly in faeces.
2. The model for lymphatic circulation was modified. A delay term, *Lymphlag* was introduced to describe a delay between DPHP entering lymphatic circulation and the subsequent appearance in venous blood at the thoracic duct. Mixing into venous blood was modelled as a first order process (proportional to the mass in lymphatic circulation). This description of the lymph in the baseline model resulted in slow emptying from the lymph into venous blood. This modification to the model was necessary in order to approximate the almost complete elimination of DPHP from blood over a 48 h period apparent in BM data.
3. A delay term, *Gutlag* was introduced to allow a delay in the uptake of DPHP from the GI tract. A better representation of the absorption phase of BM data was achieved following this modification.
4. The model was adapted to simulate the transport process of enterohepatic recirculation. Uptake of both DPHP and MPHP from the liver into bile was modelled as a first order uptake process with a delay (to represent transport from liver to gut) before DPHP (and MPHP) appeared in the gut where DPHP and MPHP were available for reabsorption (Figure 3). Data on the deposition rates of OH-MPHP and cx-MPHP in urine voids (mg/hour), calculated from the raw BM data, showed evidence of regularly spaced harmonics following the initial peak that were consistent with this process. As a consequence of this modification the PBPK model was solved as a system of delay differential equations (DDEs) rather than ordinary differential equations (ODEs).
5. First order elimination rates for DPHP and MPHP were included to account for fractions of recirculated DPHP and MPHP that were eliminated in faeces rather than reabsorbed from the GI tract.

### Parameterisation

Baseline estimates of organ and tissue masses and regional blood flows were taken from Brown et al. (1997) and ICRP (2002). The mass of the lymphatic system was obtained from Offman et al. (2016).

Tissue: blood partition coefficients were estimated using algorithms as described previously.

The bio-transformation of DPHP to MPHP in the liver was described by an intrinsic clearance term determined *in vitro* and scaled to *in-vivo*: the half-life of DPHP was estimated using the PBPK model. The *in-vivo* intrinsic clearance of DPHP in the gut was calculated using the *in vitro* hepatic clearance scaled to *in vivo* using gut microsomal protein yield and gut volume. The bio-transformation of MPHP to second order metabolites (OH-MPHP and cx-MPHP) in the liver was of the same form as the expression for DPHP, however an estimate of the half-life for MPHP was determined experimentally (as described above). A single term for metabolism of MPHP was coded in the PBPK model with the rates of removal of two direct metabolites, (OH-MPHP and cx-MPHP), from plasma assumed to be proportional to the rate of metabolism of MPHP. A urinary elimination constant was estimated for OH-MPHP and cx-MPHP.

Baseline values for parameters for which there was no prior knowledge such as *FracDOSEHep, FracDOSELymph* and the various delay terms and uptake and elimination rates were determined during the model development and testing process to provide a reasonable (but not optimised) fit to BM data.

Baseline (default) values are given in Table 3.

**TABLE 3:** Physiological and kinetic default values used in PBPK model and probability distributions applied for uncertainty and sensitivity analyses.

Physiological Parameters	Abbreviation	Default Value	Distribution
**Body weight (kg)**	BW	72.3	N^a^(72.3, 9.05)
**% BW**
Total vascularised tissues	VT	0.95	-
Liver	VLiC	3.09	N(3.09, 0.8)
Fat	VFaC	19.5	LN(3.42, 0.43)
Gut	VGuC	1.50	U(1.19, 1.84)
Stomach	VStC	0.22	N(0.22, 0.07)
Slowly perfused tissue	VSpdC	60.7	N(60.7, 9.4)
Rapidly perfused tissue	VRpdC	3.71	N(3.7, 0.26)
Blood	VBldC	5.0	U(2.5, 10)
Lymph	VLymphC	0.36	U(0.18, 0.72)
**Cardiac output (L h^−1^ kg^−1^ BW)**	QCC	14	N(13.8, 2.5)
**% Cardiac output**
Liver	QHepartC	6.0	N(6.89, 0.52)
Fat	QFaC	5.0	N(5.3, 0.3)
Gut	QGuC	14.9	U(13.2, 16.6)
Stomach	QStC	1.1	N(1.1, 0.08)
Slowly perfused tissue	QSpdC	27.0	N(28.7, 1.91)
Rapidly perfused tissue	QRpdC	42.0	N(43.1, 2.78)
Lymph	QLymphC	0.04	U(0.02, 0.08)
**Metabolic Clearance (minutes)**
In vitro half-life DPHP	T_½DPHP_	3^b^	U(15, 60)
In vitro half-life MPHP	T_½MPHP_	8.05	N(30.54, 2.39)
In vivo DPHP gut half-life	T_½DPHP_gut_	60^c^	U(15, 60)
**Elimination (gut to bowel) (h^−1^)**
DPHP	k1_DPHP_gut	0.1	U(0.05, 0.15)
MPHP	k1_MPHP_gut	0.1	U(0.05, 0.15)
**Elimination (liver to bile) (h^−1^)**
DPHP	k1_DPHP_liver	10	U(5, 15)
MPHP	k1_MPHP_liver	1	U(0.5, 1.5)
**Microsomal protein yield (mg g^−1^)**
Hepatic	MPY	34^d^	See Table 4
Gut	MPY_gut_	3.9^e^	U(1.95, 7.8)
**Fraction Bound in plasma (proportion)**
DPHP	FBDPHP	0.0025	U(10^-5^, 0.01)
MPHP	FBMPHP	0.0146	U(0.001, 0.01)
**Gastric emptying (h^−1^)^f^**
Maximum	k_(max)_	10.2	U(5.1, 20.4)
Minimum	k_(min)_	0.005	U(0.0025, 0.01)
**Absorption (h^−1^)**
Gut	k_Ga_	25.1	U(12.55, 50.2)
Time taken to consume dose (h)	DRINKTIME	0.25	U(0.125, 0.5)
Absorption in Stomach	BELLYPERM	0.685	U(0.34, 0.99)
Absorption in GI Tract	GIPERM	5.1	U(0.1, 0.3)
Absorption in Lymph via stomach	BELLYPERMLymph	0.685	U(0.34, 0.99)
Absorption in Lymph via GI Tract	GIPERMLymph	5.1	U(2.6, 7.6)
Absorption into blood from lymph	K1Lymph	0.2	U(0.1, 0.3)
Fraction of dose taken up into liver	FRACDOSEHep	0.1	See Table 4
Fraction of dose taken up into lymphatic system	FracDOSELymph	0.05	
Fraction of MPHP metabolised	FracMetab (cx and OH)		See Table 4
**Urinary elimination rate (h^−1^)**
OH-MPHP	K1_MOH	0.1	U(0.05, 0.15)
cx-MPHP	K1_cx	0.1	U(0.05, 0.15)

a Distributions, N = normal, LN = Lognormal, U = uniform

b Estimated

c Estimated

d (Bartar et al., 2007; Howgate et al., 2006)

e (Pacifici, et al., 1988; Soars, et al., 2002)

f (Loizou and Spendiff, 2004)

### Statistical Analysis

#### Parameter Distributions

Probability distributions for uncertainty and sensitivity analysis of the final PBPK model are listed in Table 3. Anatomical and physiological parameter distributions were obtained from the freely available web-based application PopGen (McNally et al., 2014). A population of 10,000 individuals comprising of 100% Caucasian males was generated. The range of ages, heights and body weights supplied as input to PopGen were chosen to encompass the characteristics of the volunteers who participated in the human volunteer study (Klein et al., 2018). Parameter ranges for organ masses and blood flows were modelled by normal or log-normal distributions as appropriate with parameters estimated from the sample and truncated at the 5th and 95th percentiles.

Uniform distributions were ascribed to the various delay terms and uptake and elimination rates. The upper and lower bounds in Table 3 were refined during the model development process. The tabulated values are therefore based upon expert judgement and represent conservative yet credible bounding estimates.

### Uncertainty Analysis

As described above, uncertainty analysis was conducted throughout the model development process in order to efficiently establish the bounding behaviour of the model (i.e. the variations in model outputs under study that were consistent with the current version of the model, and parameter value uncertainty defined through probability distributions). A 200 point maxi-min Latin Hypercube Design (LHD) was created based upon the probability distributions ascribed to model parameters and the PBPK model was run for each of these design points; the behaviour of the final model was studied based upon the probability distributions given in Table 3.

The development process followed here was broadly similar to that of (McNally et al., 2019). However whereas (McNally et al, 2019) monitored only three outputs from their PBPK model for DINCH, eight outputs from the model for DPHP were monitored in order to study the absorption, uptake, metabolism and excretion of DPHP, these were: amount of DPHP (mg) in the bowel compartment (i.e. DPHP excreted in faeces), amount of DPHP (mg) in the lymph compartment, concentrations of DPHP and MPHP in venous blood, masses of DPHP and MPHP in the plasma compartment (i.e. bound to proteins within plasma and hence unavailable for metabolism), rates of deposition of OH-MPHP and cx-MPHP in urine (mg/hour). Furthermore mass balance of DPHP and MPHP were monitored to ensure that mass balance was retained for all the tested parameter variations. The differing units of the outputs under study reflect the different aspects of model outputs that the uncertainty analysis was designed to study. This phase of work is only briefly reported on in results.

### Sensitivity Analysis

Sensitivity analysis was conducted throughout the model development process in order to study the key model output sensitivities for each version of the model under development. A two-phased GSA was implemented (McNally et al., 2011; Loizou et al., 2015) comprising of elementary effects screening (Morris Test) followed by a variance-based approach. Results from sensitivity analysis for the final model were obtained using the probability distributions given in Table 3.

A total of 59 parameters were varied in elementary effects screening, with five elementary effects per input computed, leading to a design of 300 runs of the PBPK model. The model outputs studied are described below.

Concentrations of DPHP and MPHP in venous blood at 0.5, 3 and 12 h and 1, 3 and 12 h following ingestion, respectively were studied using elementary effects screening. The three output times studied were broadly of representative of the following periods in the concentration-time courses: prior to peak concentration (of DPHP and MPHP); post peak concentration; and returning to baseline (zero) concentrations. Rates of deposition of OH-MPHP and cx-MPHP in urine were studied at 3-, 12- and 20-h following ingestion for both model outputs with these times corresponding to the periods where peak concentration in urine was reached; when the first harmonic (due to `hepatic recirculation) was predicted (12 h); and returning to baseline. Finally, the concentrations of DPHP and MPHP in plasma were studied. Rather than studying model output at specific time points, instead the peak concentrations of DPHP and MPHP in plasma; the times that corresponded to these peak concentrations; and the rate of change of DPHP and MPHP in plasma over the hour following the peak concentrations, were extracted from each of the 300 model runs. These measures were chosen since they proved to be more useful metrics for understanding the BM data of Klein et al. (2018), and in particular the more rapid clearance from plasma than would be expected given the predictions of logP (and from this an estimate of protein binding) (McNally et al., 2019). This phase of sensitivity analysis is only briefly described in results.

All parameters that were within 0.2 of the maximum value of µ* (one of the two measures computed in elementary effects screening) for any of the 18 metrics studied (three metrics for each of the six outputs described above) were retained and studied in the second phase of analysis using the variance-based analysis.

In the second phase of analysis 31 retained parameters were studied using the extended Fourier Amplitude Sensitivity Test (eFAST). In this analysis 1,000 runs per retained parameter were conducted, leading to 31,000 simulations of the PBPK model. In this more computationally expensive phase of sensitivity analysis, the rates of deposition of OH-MPHP and cx-MPHP in urine and concentrations of DPHP and MPHP in plasma were studied.

### Calibration

Calibration of a subset of sensitive model parameters using the BM data of (Klein, et al., 2018) was attempted. A Bayesian approach was followed (McNally et al., 2012). This requires the specification of a joint prior distribution for the parameters under study, which is refined through a comparison of PBPK model predictions and measurements within a statistical model. The resulting (refined) parameter space that is consistent with the prior specification and measurements is the posterior distribution.

The final calibration model utilised data from five of the six individuals (data from individual E were unusual and thus excluded) from the BM study of (Klein et al., 2018) with data on four specific outputs formally compared within the calibration model. Concentrations of DPHP and MPHP (*CBlood DPHP* and *CBlood MPHP*) and the rates of deposition of OH-MPHP and cx-MPHP (mg/hour) into the bladder (*RUrine OH* and *RUrine* cx) were computed from the raw data (Klein et al., 2018). The latter measure i.e. the concentration (mg/L), the volume of the void (ml) and the times between voids (hours)) – (see for example Nehring et al. (2020) represents the underlying trends in concentration response data in a more precise manner than expressing the metabolite concentration relative to creatinine concentration. These measurements were compared to corresponding predictions from the PBPK model using the statistical models depicted in Eqs. 7–10.

The terms RUrineOHij,RUrinecxij,CBloodDPHPij and CBloodMPHPij denote measurement i (at time ti) for individual j (for j in 1:5) for the four respective model outputs, whereas $\mu_{OH}{\left(\theta ,\omega_{j}\right)}_{ij}$, $\mu_{\text{CX}}{\left(\theta ,\omega_{j}\right)}_{ij}$, $\mu_{DPHP}{\left(\theta ,\omega j\right)}_{ij}$and $\mu_{MPHP}{\left(\theta ,\omega_{j}\right)}_{ij}$denote the predictions from the PBPK model corresponding to parameters $\left(\theta ,\omega_{j}\right)$. The vectors θ and $\omega_{j}$ denote the global parameters common to all individuals (suitable for partition coefficients, fractions metabolised to OH-MPHP and cx-MPHP etc.) and participant specific parameters (suitable for accounting for variability in the physiology and modelling the participant specific uptake of DPHP etc.). $\sigma_{OH},\sigma_{CX},\sigma_{DPHP}$ and $\sigma_{MPHP}\;$ denote the respective error standard deviations. Normal distributions, truncated at zero were assumed for all four relationships.

$$RUrineOH_{ij}∼N\left(\mu_{OH}{\left(\theta ,\omega_{j}\right)}_{ij},\sigma_{OH}\right)\left[0,\infty \right]$$ (7)

$$RUrinecx_{ij}∼N\left(\mu_{cx}{\left(\theta ,\omega_{j}\right)}_{ij},\sigma_{cx}\right)\left[0,\infty \right]$$ (8)

$$CBloodDPHP_{ij}∼N\mu_{DPHP}{\left(\theta ,\omega_{j}\right)}_{ij},\sigma_{DPHP}\left[0,\infty \right]$$ (9)

$$CBloodMPHP_{ij}∼N\left(\mu_{MPHP}{\left(\theta ,\omega_{j}\right)}_{ij},\sigma_{MPHP}\right)\left[0,\infty \right]$$ (10)

Prior distributions for global and local parameters in the PBPK model were taken from Table 3 (for the sensitive parameters that were studied). Non-informative gamma (0.01, 0.01) prior distributions were assumed for the four standard deviation parameters.

Inference for the model parameters was made using Markov chain Monte Carlo (MCMC) implemented in MCSim (see Software). Inference for model parameters in the final calibration model was made using thermo-dynamic integration (TI) as described in Bois et al. (2020). A single chain of 1,000,000 iterations was run with every 10th retained.

### Software

The PBPK model was written in the R language and run using the RStudio and RVis software applications during the development of the PBPK model. PBPK models were solved using the deSolve package of R. The DiceDesign package of R was used for generating Latin Hypercube designs. GSA of model outputs (through elementary effects screening and eFAST) were conducted using the Sensitivity package of R. The reshape2 package of R was used for reshaping of data for plotting and other processing of results.

The PBPK model was rewritten in the GNU MCSim language prior to calibration. MCMC was undertaken using the TI option within GNU MCSim

All plots were created using R and the gg2plot package.

## Results

### Uncertainty and Sensitivity Analysis

**FIGURE 4:**
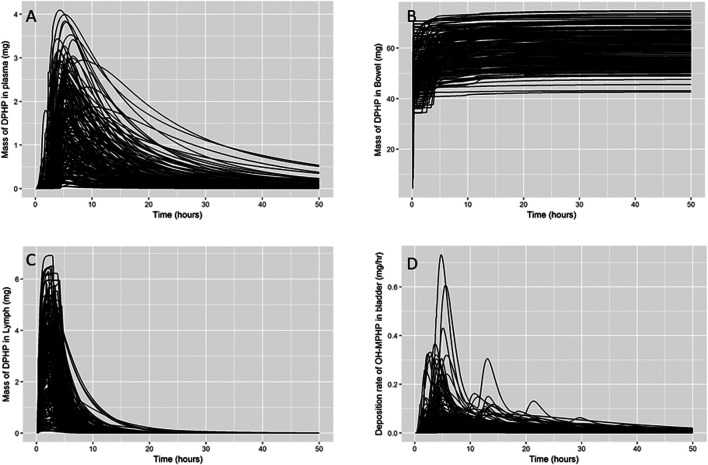
Uncertainty analysis. Variability in the mass of DPHP in plasma (mg) **(A)**, bowel (mg) **(B)** and lymph (mg) **(C)** and variability in predicted rates of elimination of OH-MPHP in urine (mg/hr) **(D)**.

In Figure 4 a small subset of results from uncertainty analysis of the final model are shown: each curve shows the predictions of a particular model output corresponding to a design point. These plots indicate that the model structure remains stable over a wide range parameter values. Figures 4A–C show the mass of DPHP within the plasma, lymph and bowel compartments and were used to study the range of behaviours of specific aspects of the model that could be achieved based upon the form of the PBPK model and through variations in the uncertain parameters. Through this uncertainty analysis of the mass of DPHP in plasma (Figure 4A) the effect of protein binding on the mass retained and subsequent elimination from plasma could be studied. Specifically, in this exploratory phase of work we had a particular interest in attempting to replicate the unusual findings from (Klein et al., 2016), who reported peak concentrations of DPHP in the blood of volunteers which occurred later than the corresponding peak of the metabolite MPHP in blood and also later than peak concentrations of second order metabolites in urine. Through uncertainty analysis of DPHP in the lymph, variability in the uptake, retention and elimination of DPHP in the lymph compartment could be studied. The uncertainty analysis of DPHP in the bowel allowed the study of variability in the fraction of DPHP that was initially unabsorbed and the further removal of DPHP following elimination in bile. Figure 4D shows predictions of the rate of elimination of OH-MPHP in urine (mg/hr), one of the chosen metrics for calibration, and was studied to establish how well the unique trends in urine voids from the six volunteers could be captured by the final model. Other checks on a range of outputs or functions of model outputs (masses, rates and concentrations) were also undertaken in this phase of modelling to ensure behaviour of the model appeared reasonable over the range of parameter space specified through probability distributions.

**FIGURE 5:**
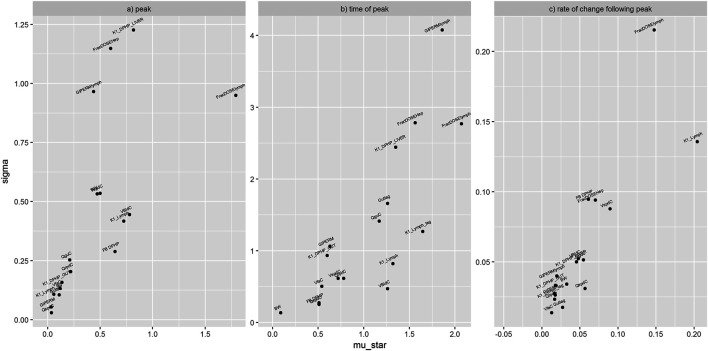
Elementary effects screening (Morris Test) of the mass of DPHP in plasma. Sensitivity of the peak mass of DPHP in plasma (mg) **(A)**, the time when peak concentration was reached (hours after dosing) **(B)** and the rate of change of DPHP in plasma (mg/hour) in the hour following peak concentration **(C)**. Areas with overlapping parameter labels represent clusters of parameters with minimal sensitivity.

Figure 5 shows the results from elementary effects screening (Morris Test) applied to the mass of DPHP in plasma. A high μ* indicates a factor with an important overall influence on model output; a high σ indicates either a factor interacting with other factors or a factor whose effects are non-linear. The magnitude of μ* and σ for each model parameter is relative, i.e., a parameter has a low μ* relative to the parameter with the highest μ*. Results from this technique are usually obtained at specific time points, i.e. sensitivity analysis of a given model output at say, 1 h following dosing. However sensitivity analysis can be applied to any chosen model outputs calculated from each model run specified through the design. The results shown Figure 5 correspond to some unusual measures calculated from model output that were chosen to study particular aspects of model behaviour: sensitivity analysis of the peak mass of DPHP in plasma (mg) (A), the time when peak concentration was reached (hours after dosing) (B) and the rate of change of DPHP in plasma (mg/hour) in the hour following peak concentration (C). The parameters with lower overall importance are clustered toward zero of both axes. Unfortunately, we could not prevent the overlapping of some parameter labels in this region (Figure 5).

The parameters ranked as most important by the Morris test were analysed by eFAST. The period from the start of the simulation to 20 h showed the most variance in blood concentrations of DPHP and MPHP. The most important parameters were reduced further in number and ranked as follows: FBMPHP, FBDPHP, FracDoseLymph, FracDoseHep, Lymphlag, PbaM, QCC, K1Lymph, VspdC, VliC, QguC, and VbldC. The fractions of MPHP and DPHP bound to plasma proteins were significantly more important than the other parameters over this period.

The period from the start of the simulation to 25 h showed the most variance in urinary excretion of OH-MPHP and cx-MPHP. The most important parameters were ranked as follows: FracDoseHep, FracMetabMOH, FracMetabcx, BW, K1_MOH, K1_cx, QCC, GIPERM1, PguM, QguC, PliM, and PbaM. The first four parameters were significantly more important for variance in urinary excretion than the other parameters over this period.

### Calibration

**FIGURE 6:**
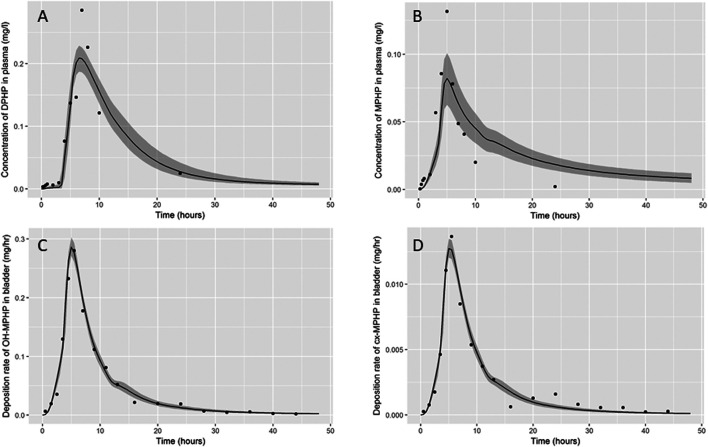
Fit of the calibrated model to data **(A)** blood DPHP, **(B)** blood MPHP and urinary excretion of **(C)** OH-MPHP and **(D)**_cx-MPHP for volunteer A using global (non-volunteer specific) and local (volunteer specific) parameters provided in Table 2, Table 3, and Table 4. The central estimates indicated in plots correspond to the posterior mode whereas the shaded regions represent 95% intervals for the respective curves.

**FIGURE 7:**
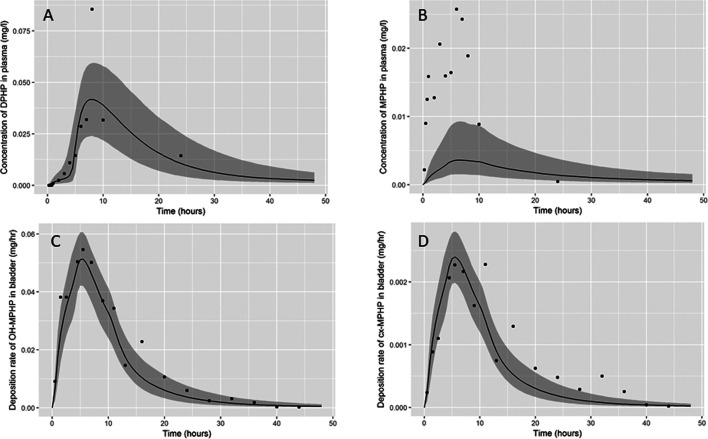
Fit of the calibrated model to data **(A)** blood DPHP, **(B)** blood MPHP and urinary excretion of **(C)** OH-MPHP and **(D)** cx-MPHP for volunteer C using global (non-volunteer specific) and local (volunteer specific) parameters provided in Table 3 and Table 4. The central estimates indicated in plots correspond to the posterior mode whereas the shaded regions represent 95% intervals for the respective curves.

**FIGURE 8:**
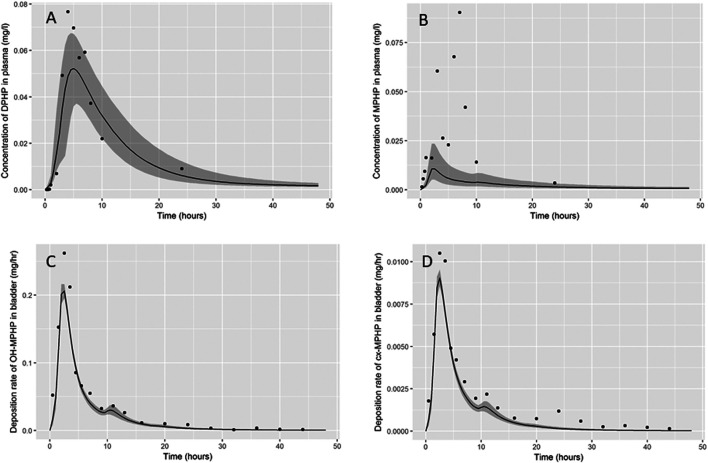
Fit of the calibrated model data **(A)** blood DPHP, **(B)** blood MPHP and urinary excretion of **(C)** OH-MPHP and **(D)**_cx-MPHP for volunteer D using global (non-volunteer specific) and local (volunteer specific) parameters provided in Table 3 and Table 4. The central estimates indicated in plots correspond to the posterior mode whereas the shaded regions represent 95% intervals for the respective curves. The trends for volunteer D were broadly representative of volunteers B and F whose data are not shown.

Summary statistics based upon the retained sample (posterior median and a 95% credible interval) for the global and local (volunteer specific) parameters are provided in Table 3 and Table 4 respectively. The fit of the calibrated model is shown in Figures 6A–D, Figures 7A–D and Figures 8A–D for three of the five participants. The trends for the individual shown in Figure 8 are broadly representative of the two individuals whose data are not shown. The central estimates indicated in plots correspond to the posterior mode whereas the shaded regions represent 95% intervals for the respective curves. This is a pointwise credible interval which was derived through running the PBPK model for each retained parameter set, ordering the predictions by magnitude at each time point and reading off the 2.5^th^ and 97.5^th^ percentiles.

**TABLE 4:** Summary statistics from marginal posterior distributions for calibrated global parameters.

Parameter	Posterior median (95%credible interval)
FB_DPHP	0.991 (0.989, 0.995)
FB_MPHP	0.975 (0.956, 0.986)
DPHP_Gut_half_life	56.80 (46.96, 59.90)
DPHP_half_life	4.38 (3.05, 9.10)
Pbab	22.92 (9.10, 29.70)
Pgub	20 (14.20, 26.98)
Plib	4.22 (1.14, 23.14)
PbaM	31.84 (8.37, 49.17)
PliM	14.92 (1.15, 48.51)
PguM	38.57 (24.70, 49.33)
K1_MOH	0.973 (0.87, 0.99)
K1_cx	0.778 (0.67, 0.89)
FracMetab_OH	0.24 (0.21, 0.31)
FracMetab_cx	0.011 (0.01, 0.014)
K1_DPHP_Liver	3.09 (0.1, 16.9)
K1_MPHP_Liver	0.9 (0.02, 10.9)
K1_DPHP_Gut	0.3 (0.04, 0.5)
K1_DPHP_Gut	0.4 (0.07, 0.5)
$\sigma_{OH}$	0.01 (0.008, 0.012)
$\sigma_{cx}$	0.0005 (0.0004, 0.0006)
$\sigma_{DPHP}$	0.018 (0.04, 0.024)
$\sigma_{MPHP}$	0.019 (0.016, 0.024)

The BM data from each of the study participants showed unique trends. The blood DPHP data indicated differences in lag prior to uptake, rates of uptake and fractions absorbed from the gut. In the blood data of some participants, the first order metabolite MPHP was measurable in blood prior to the appearance of parent chemical (Figure 6A and Figure 7A). Uptake of DPHP appeared to be multi-phased in some participants. The PBPK model contained nine parameters that were tuned to the BM data from each participant for fitting the hepatic uptake of DPHP and a further three parameters governing uptake into the lymphatic system and subsequent deposition into blood at the thoracic duct (Table 3). The results in Figures 6–8 demonstrate that the BM data on DPHP in blood were captured by the calibrated model. The differences in the calibrated individual specific parameters (Table 3) reflect the large differences in the trends of DPHP in blood for the study participants.

In contrast, the rate of deposition of second order metabolites in urine was mainly governed through global (non-volunteer specific) parameters (Table 4). Reasonable fits were obtained for the urinary excretion of OH-MPHP and cx-MPHP with the model successfully fitting earlier peaks in the urine compared to blood. Enterohepatic recirculation of DPHP was an important mechanism which is observed in the harmonics seen in the OH-MPHP and cx-MPHP time courses at intervals of approximately 8 h post peak concentration (Figures 6C,D and Figures 8C,D). The fit to the data shown in Figures 7C,D was poorer and appears to be consistent with a second absorption event (not explicitly captured in the model). Data from another volunteer, not used in final calibration, showed even stronger evidence of a second absorption event. As enterohepatic recirculation was modelled using global parameters, the differences seen in the simulations of urine data for these three participants appear to arise as a consequence of differences in uptake of DPHP.

The fits to MPHP in blood were generally quite poor; particularly when MPHP spiked in blood specimens shortly after DPHP was consumed by study participants. This highlights a deficiency in the model, which does not impact upon the ability of the model to accurately predict deposition of second order metabolites in urine. We address this deficiency further in the discussion section.

The bound fractions of DPHP and MPHP were 0.991 (0.989, 0.995) and 0.975 (0.956, 0.986), which represent very high binding in blood, although somewhat smaller than the very high fractions predicted by algorithms. There are no known direct measurements of protein binding of DPHP or MPHP in blood although there are estimates of the “free” area-under-the curve (AUC) concentrations of MPHP as a proportion of total DPHP concentration which are approximately 66% (Klein et al., 2018). The half -life of DPHP, which could not be estimated in vitro incubations, was estimated in the model to be very short in the liver at 4.38 min (3.05, 9.10), and approximately a factor of 10 greater than in the gut 56.80 min (46.96, 59.90).

The vast majority of DPHP was unabsorbed from the gut. Total uptake ranged from 16.3% (13.1%, 19.3%) to 2% (1.5–2.6%), dominated by hepatic uptake at around a factor of five greater than lymphatic uptake. The fractions of absorbed DPHP excreted as OH-MPHP and cx-MPHP were estimated as 0.24 (0.21, 0.31) and 0.011 (0.01, 0.014).

**FIGURE 9:**
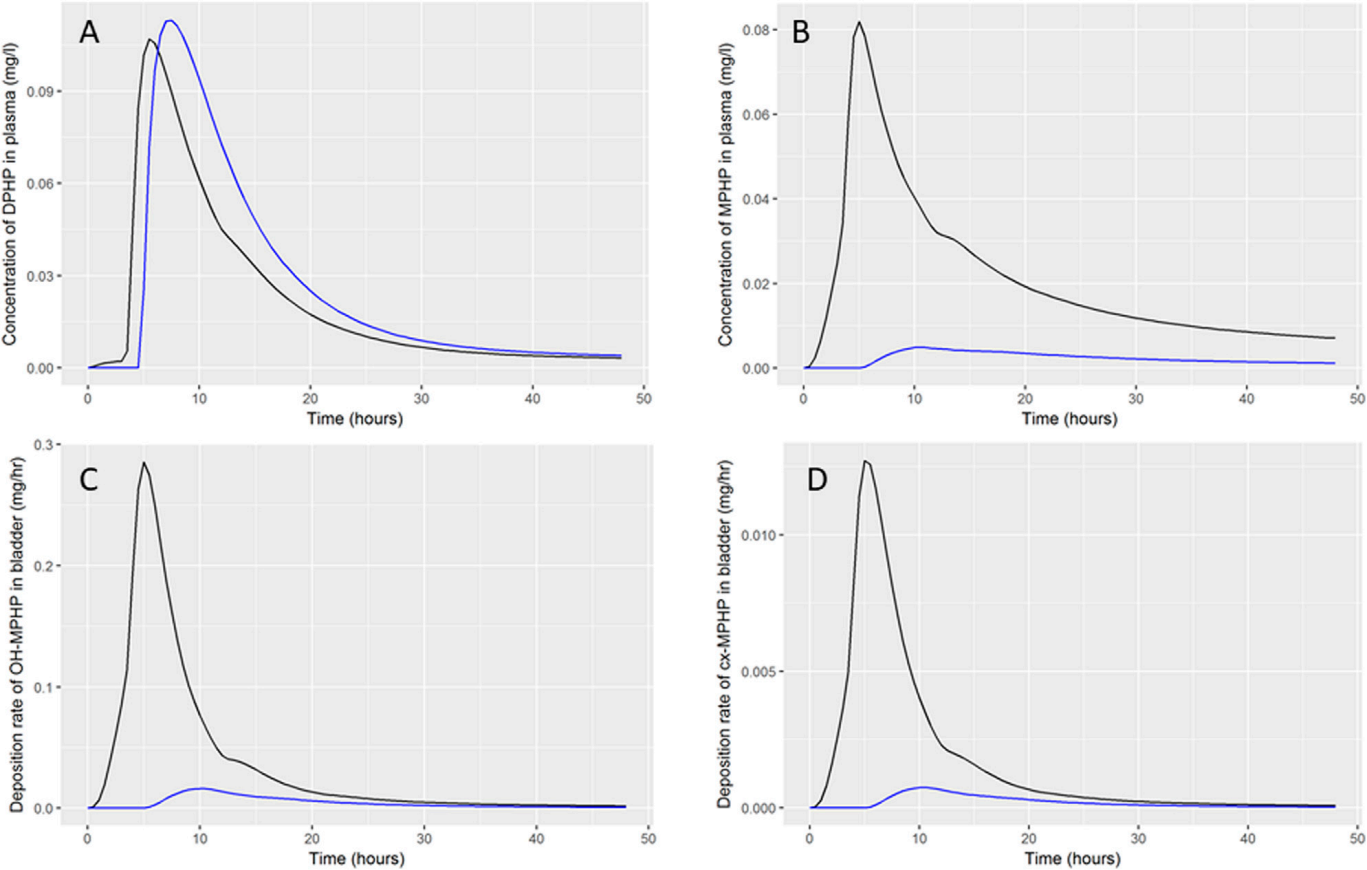
Comparison of DPHP hepatic and lymphatic routes of uptake. Simulations were based upon optimised parameters for individual A but where the lymphatic fraction (black lines) or the hepatic fraction (blue lines) was set to zero.

A comparison of simulations of entry of DPHP through the hepatic (black line) and lymphatic (blue line) routes is shown in Figure 9. These simulations were based upon optimised parameters for individual A where with lymphatic fraction was set to zero (black lines) or the hepatic fraction set to zero (blue lines). Thus, the independent effects of absorption through the two routes at otherwise credible values for model parameters could be studied. The key biological difference between the two routes of entry is that DPHP absorbed through the hepatic route is subject to first pass metabolism, primarily in the liver, whereas the lymphatic fraction by-passes first pass metabolism. Despite the hepatic fraction being a factor of five greater, the peak plasma concentration from the two routes was similar (Figure 9A), which indicates that a very large fraction of DPHP absorbed through the hepatic route is intercepted by the liver and never enters systemic circulation. Uptake of DPHP into the systemic circulation from either hepatic or lymphatic routes is almost completely bound to proteins in plasma and is thus neither available for distribution to organs and tissues nor for metabolism. Our simulations indicate that DPHP entering the systemic blood circulation via the lymphatic route is almost entirely held in plasma and is important for understanding trends in plasma. It does however represent a small mass of DPHP, which has a negligible impact on trends in MPHP in blood and second-order metabolites in urine. In particular, due to the high binding in plasma and the consequent reduced rate of metabolism only a shallow peak with a long tail can be seen in urinary metabolite simulations.

## Discussion

In this study we supplement the interpretation of the data of (Klein et al., 2018) with additional insights using a PBPK model calibrated for DPHP kinetics.

Only a minority fraction of ingested DPHP was absorbed from the gut. The model suggested the fraction of absorbed DPHP (quantified as the complement of the fractions entering lymphatic and hepatic circulation respectively) ranged from 2% (1.5%–2.6) to 16.3% (13.1%, 19.3%) for the six study participants. In addition to a substantial variation in the fraction absorbed between participants, there was also a substantial variation between participants in the rate of absorption, quantified through the *Gutlag* and *GIperm* parameters. The PBPK model contains only a simple description of the gut, with modelling via a single compartment. More detailed models of the GI tract, such as the ACAT (Gobeau et al., 2016) model, describe the gut as a series of linked compartments, each with their own permeability and pH. The *Gutlag* in our PBPK model can be interpreted as a delay until parent chemical reaches a section of the GI tract where absorption occurs. Klein et al. (2018) provided detailed information on the food consumed by individual study participants and the time of consumption relative to the ingestion of DPHP. This relatively uncontrolled aspect of the human volunteer study appears to have contributed, in addition to the measured volunteer specific parameters (Table 2) to the large inter-individual variability in BM data.

Some features of the BM data are difficult to interpret, such as the time to peak concentrations of second order metabolites in urine occurring prior to the time to peak of DPHP concentration in blood, which is counter-intuitive. However, deeper insights into the pharmacokinetics of DPHP in humans are possible through the development of a PBPK model. Our model suggested that the majority of the absorbed fraction of DPHP entered via the hepatic route. Metabolism of DPHP was primarily in the liver, and following eventual absorption this was rapid. A fraction of DPHP was transported from the liver tissue to the gut in bile. The rapid elimination of DPHP is explained by first pass metabolism and biliary excretion. Only traces of parent chemical and first order metabolite appeared to enter into the systemic circulation. A small fraction of DPHP (approximately 20% of that entering via the hepatic route) entered the systemic circulation via the lymphatic route. A detailed description of lymphatic flow was not described in the model: instead the simplified process of absorption into the lymph compartment and appearance in venous blood at a rate proportionate to the amount in the lymph, following a lag time in hours (*Lymphlag*), was described. DPHP entering via this route avoided first pass metabolism and thus entered the systemic circulation. Binding of DPHP (approx. 99%) was high although notably lower (a factor of 10) than the extreme value predicted by the predictive algorithms (Table 1). DPHP entering via the lymph was almost entirely held within the plasma compartment until stripped from proteins and metabolized and as a consequence this minor absorption route had a significant influence on DPHP in plasma. The delayed peak of DPHP concentration in venous blood (relative to MPHP in blood and second order metabolites in urine) can be explained as a result of three processes: 1) DPHP entering the systemic circulation from the lymph, 2) rapid and very high protein binding and 3) the efficiency of the liver in removing DPHP absorbed via the hepatic route.

MPHP concentration peaked prior to DPHP concentration in blood specimens for five of the six volunteers. In some volunteers MPHP spiked rapidly and could be detected in blood prior to DPHP. Through the inclusion of a two compartment gut and a description of metabolism of DPHP in the gut we were able to jointly model DPHP and MPHP in blood, however it was not possible to fit MPHP in blood once data from urine specimens were also used in calibration; the very early peak of MPHP observed in blood did not result in early peaks of OH-MPHP and cx-MPHP in urine. This inconsistency would indicate a deficiency in our model. This could potentially be explained if MPHP absorbed through the gut was bound within plasma and thus unavailable for metabolism. In the PBPK model, binding in arterial blood was described however, MPHP absorbed in the gut was fully available for first pass metabolism prior to binding. A better fit to blood MPHP may potentially be achieved by describing very rapid plasma binding following absorption of MPHP in the gut. This possible change to metabolism of DPHP in the gut and the subsequent absorption of MPHP would not affect the fit to urinary metabolite data since the vast majority of metabolism of DPHP and MPHP occurs very rapidly in the liver. The biological plausibility of this mechanism would require investigation and its importance to the risk assessment of DPHP confirmed to justify subsequent modification of the model.

We consider the measurements of OH-MPHP and cx-MPHP in urine to provide the most reliable guide of the fate of DPHP following ingestion. These data indicate that parent chemical was absorbed from the gut and rapidly metabolised, with metabolism almost entirely occurring within the liver, and with further rapid metabolism of MPHP in the liver. Only traces of DPHP and MPHP appear to have entered into the systemic circulation. Elimination of second order metabolites OH-MPHP and cx-MPHP from blood was rapid. The urine samples provided evidence of enterohepatic recirculation with up to three visible harmonics following peak exposure, at intervals of approximately 8 hours; this is a new insight from our modelling that was not discussed by Klein et al. (2018). The evidence for the lymphatic route was weaker from urinary metabolite data, since binding of DPHP and MPHP results in a prolonged elimination process, and the fraction entering via the lymphatic route is modest compared to the hepatic route. Elimination of secondary metabolites of DPHP is increased over a 24-h period compared to a model that does not account for this route. (Klein et al., 2018) interpreted the first harmonic of secondary metabolites in urine specimens as evidence of the lymphatic route, however our simulations indicate that a sharp peak could not be achieved from lymphatic uptake, in contrast, enterohepatic recirculation could account for such sharp peaks. We are unaware of previous work that has suggested strong evidence of enterohepatic recirculation from urinary metabolite measurements.

An important finding of this work is that the BM data from venous blood provide an incomplete picture of the kinetics of DPHP, and a model built and calibrated to these data alone would be a poor description of the biology – such a model would be tuned to the minor absorption route. For extremely lipophilic substances like DPHP and other plasticizers (di-(isononyl)phthalate, cyclohexane-1,2-dicarboxylic acid, di(isononyl) ester etc.) the established paradigm of development and calibration of a PBPK model based upon animal (rat) data, and extrapolation to the human can be problematic – the rat study of Klein et al. (2016) only studied DPHP and its metabolites in blood and thus did not obtain the most useful data (from urine specimens) for understanding the biological mechanisms. Similarly, for this class of chemicals an incomplete picture may be obtained from an analysis of only urine specimens in the absence of blood.

Results from uncertainty and sensitivity analyses of the final model for DPHP only were presented in this work. However, uncertainty analysis conducted using Latin Hypercube sampling and GSA (using elementary effects screening and eFAST) were utilized iteratively at various phases as the model for DPHP was being developed. Although these techniques cannot inform which biological processes may be missing from a model in development, they can quickly identify the bounding behaviour of the current version of the model and identify the key uncertain parameters that drive variability observed in dose-response in simulations. These techniques proved to be invaluable in developing, debugging and understanding a complex PBPK model. The overall framework of uncertainty and sensitivity analysis followed in this work replicates that of McNally et al. (2019), however some of the metrics studied using GSA (the timing of a peak concentration and the rate of change following the peak) are novel. This work highlights the flexibility of the GSA techniques and demonstrates that through correct application of high level analyses within a model development framework, an experienced modeller may make insights about the behaviour of the model and thus the underlying biology to narrow the research space and guide targeted future experimental evaluations.

Development of the model was initially in R syntax with a system of DDE’s solved using the deSolve package. The R language offers a flexible framework for the specification of PBPK models and the deSolve package offers a wide range of solvers. Whilst this modelling environment proved to be suitable for development of the model and uncertainty and sensitivity analysis, initial attempts at calibration demonstrated that R was too slow for the calibration of DDE’s. The PBPK model was therefore rewritten in GNU MCSim, a more suitable language for intensive computations. Calibration was conducted using the thermodynamic integration variant of MCMC (Bois et al., 2020).

## Data Availability

The raw data supporting the conclusions of this article will be made available by the authors, without undue reservation.

## Footnotes

1
https://eur-lex.europa.eu/legal-content/EN/TXT/HTML/?uri=OJ:L:2011:012:FULL&from=FR

2 Concentration in human biological material at which, and below which, there is no risk of adverse health effects
