# Supplementary material for: Development, Testing, Parameterisation and Calibration of a Human PBPK Model for the Plasticiser, Di-(2-propylheptyl) Phthalate (DPHP) Using in Silico, in vitro and Human Biomonitoring Data
Source: Front Pharmacol. 2021 Sep 2;12:692442. doi: 10.3389/fphar.2021.692442 (PMC8443793; doi:10.3389/fphar.2021.692442)
Supplement: Supplementary file 1 [file DataSheet1.docx]

# Supplementary Materials

Development, testing, parameterisation and calibration of a human PBPK model for the plasticiser, Di-(2-propylheptyl) phthalate (DPHP) using in silico, in vitro and human biomonitoring data

Kevin McNally^a^, Craig Sams^a^, Alex Hogg^a^, Annie Lumen^b^ and George Loizou^a^

## Model Code in MCSim Syntax

# MCSim 5.x script

# *** DPHP ***

# Di(2-propyl heptyl) phthalate

# Kevin McNally, George Loizou, Alex Hogg (HSE) 2020-01-16T00:00:00.0000000Z

# Compiled on: 2020-01-16T15:34:16.8794082Z

States =

{

Afa,

Agu1,

Agu2,

AMgu,

Ast,

Arpd,

Aspd,

Ali,

AMli,

ABile,

Alymph,

ARBC_DPHP,

Aplasm_DPHP,

ABellyH,

AGiTractH,

ABowel,

ABellylymph,

AGiTractlymph,

AfaM,

AstM,

AguM,

AMliM,

AliM,

ABileM,

ABowelM,

AspdM,

ArpdM,

Aplasm_MPHP,

ARBC_MPHP,

AMMPHPB_MOH,

AMMPHPB_cx,

AMMPHPU_MOH,

AMMPHPU_cx,

VBladder,

Gutswitch,

Lymphswitch,

DOSESTEP

};

Outputs =

{

Cli,

Cfa,

Cgu1,

Cgu2,

Cst,

Clymph,

Cspd,

Crpd,

mass,

Uptake,

reloral,

CVfa,

CVgu1,

CVgu2,

CVst,

CVspd,

CVli,

CVrpd,

CV,

CVnmol,

CA_DPHP,

CAT_DPHP,

CRBC_DPHP,

CV_total_nmol,

CVM,

CliM,

CVliM,

CguM,

CVguM,

CstM,

CVstM,

CfaM,

CVfaM,

CspdM,

CVspdM,

CrpdM,

CVrpdM,

CA_MPHP,

CAT_MPHP,

CRBC_MPHP,

CV_total_MPHP_nmol,

relMPHP,

massMPHP,

Curine_MOH,

Curine_cx,

ODOSEliver,

ODOSElymph,

ODOSEbowel,

ClintDPHP,

ClintDPHPgu,

ClintMPHP,

Ali_lag,

AliM_lag,

Plasma_DPHP,

Plasma_MPHP,

Urine_cx,

Urine_OH

};

Inputs =

{

events_Gutswitch,

events_Lymphswitch,

events_DOSESTEP,

events_VBladder,

events_AMMPHPU_MOH,

events_AMMPHPU_cx

};

# Parameters

# ==========

#

BW = 89; # body mass (kg)

MWDPHP = 446.67; # DPHP molecular mass (g/mol)

MWMPHP = 306.41; # MPHP molecular mass (g/mol)

MWMPHPOH = 322.39; # OH-MPHP molecular mass (g/mol)

MWMPHPcx = 336.37; # cx-MPHP molecular mass (g/mol)

CAE = 0.75; # cardiac allometric exponent

QCC = 11.22; # cardiac allometric constant (L/h/kg^CAE)

VT = 0.95; # proportion of vascularised tissue

VfaC = 0.195; # fractional volume

VguC = 0.067; # fractional volume

VstC = 0.0158; # fractional volume

VspdC = 0.4714; # fractional volume poorly perfused

VrpdC = 0.033; # fractional volume richly perfused

VliC = 0.0203; # fractional volume

VlymphC = 0.0036; # lymph system fractional volume

VBldC = 0.05; # blood fractional volume

QhepartC = 0.06; # hepatic artery fractional blood flow

QguC = 0.17; # fractional blood flow

QstC = 0.01; # fractional blood flow

QspdC = 0.27; # overall fractional blood flow to slowly perfused tissue

QrpdC = 0.42; # overall fractional blood flow to rapidly perfused tissue

QfaC = 0.05; # fractional blood flow

FracDOSELymph = 0.05; # Fraction of dose taken into lymph

FracDOSEHep = 0.1; # Fraction of dose taken into hepatic

FracMetabMOH = 0.3; # Fraction of CYP-mediated metabolism MPHP -> MPHP (Table 2 Koch et al (2013) Arch Tox 87) 0.25

FracMetabcx = 0.05; # Fraction of CYP-mediated metabolism MPHP -> MPHP (Table 2 Koch et al (2013) Arch Tox 87) 0.25

FB_DPHP = 0.9975; # Fraction of DPHP bound to plasma proteins 0.9998752

FB_MPHP = 0.9854; # Fraction of MPHP bound to plasma proteins 0.9854

PORALDOSE = 0.7; # oral dose [mg/kg]

DRINKTIME = 0.25; # Drink time [h]

BELLYPERM = 0.685; # [/h]

GIPERM1 = 5.1; # [/h]

GIPERM2 = 5.1; # [/h]

BELLYPERMlymph = 0.685; # [/h]

GIPERMlymph = 5.1; # [/h]

KEMAX = 10.2; # [Maximum emptying rate /h]

KEMIN = 0.005; # [Minimum emptying rate /h]

KA_MPHP = 0.3; # 1st-order oral uptake rate of MPHP (1/hr)

Lymphswitch = 1;

Gutswitch = 1;

MPY = 34; # microsomal protein yield [mg microsomal protein/g liver]

MPYgu = 3.9; # microsomal protein yield [mg microsomal protein/g gut]

Incub_vol = 1; # Volume of incubation (ml)

Microsome_prot = 0.5; # microsomal protein amount (mg)

DPHP_half_life = 3; # DPHP -> MPHP half-life (minutes)

DPHP_GUT_half_life = 60; # DPHP -> MPHP GUT half-life (minutes)

MPHP_half_life = 8.0486; # MPHP -> OH-MPHP and cx-MPHP half-life (minutes)

RUrine = 0.1; # Rate of Urine Production [l/h]

Creat = 1.217; # Urinary creatinine concentration [g/L] or 0.01192 [mol/L]

K1_MOH = 0.1; # First-order elimination rate from blood [/h]

K1_cx = 0.1; # First-order elimination rate from blood [/h]

K1_DPHP_GUT = 0.1; # First-order elimination rate of DPHP from gut into bowel [/h]

K1_DPHP_LIVER = 10; # First-order elimination rate of MPHP from liver into bile [/h]

K1_MPHP_GUT = 0.1; # First-order elimination rate of MPHP from gut into bowel [/h]

K1_MPHP_LIVER = 1; # First-order elimination rate of MPHP from liver into bile [/h]

K1Lymph = 0.2; # First-order elimination rate from Lyph into blood [/h]

Lymphlag = 3.01; # Lag between uptake into Lymph and emptying into blood [h]

Gutlag = 3.01; # Lag between uptake into GItract and emptying into gut [h]

Pbab = 3.01; # DPHP blood:air partition coefficient

Pfab = 63.38; # DPHP tissue:blood partition coefficient

Pgub = 7.4; # DPHP tissue:blood partition coefficient

Pstb = 7.4; # DPHP tissue:blood partition coefficient

Prpdb = 3.7; # DPHP tissue:blood partition coefficient

Pspdb = 3.29; # DPHP tissue:blood partition coefficient

Plib = 5.89; # DPHP tissue:blood partition coefficient

PbaM = 6.67; # MPHP Red blood cells:plasma partition coefficient

PspdM = 7.51; # MPHP Slowly perfused tissue:air partition coefficient

PliM = 54.8; # MPHP tissue:air partition coefficient

PrpdM = 12.20; # MPHP Richly tissue:air partition coefficient

PfaM = 29.10; # MPHP Fat tissue:air partition coefficient

PstM = 25.2; # MPHP Stomach tissue:air partition coefficient

PguM = 25.2; # MPHP GI Tract tissue:blood partition coefficient

Vfa = 0;

Vgu = 0;

Vst = 0;

Vspd = 0;

Vrpd = 0;

Vli = 0;

Vlymph = 0;

Qfa = 0;

Qgu = 0;

Qst = 0;

Qrpd = 0;

Qspd = 0;

Qli = 0;

QCMC = 0;

ODOSEliver = 0;

ODOSElymph = 0;

ODOSEbowel = 0;

Uptake = 0;

Vplas = 0;

VRB = 0;

Vbld = 0;

Qhepart = 0;

CA_DPHP = 0;

CAT_DPHP = 0;

CA_CRBC = 0;

CVnmol = 0;

CV_total_nmol = 0;

CVM = 0;

CA_MPHP = 0;

CAT_MPHP = 0;

CRBC_MPHP = 0;

CV_total_MPHP_nmol = 0;

# SD terms for MCMC

# ==========

#

Sigma1 = 0.1;

Sigma2 = 0.1;

Sigma3 = 0.1;

Sigma4 = 0.1;

Initialize

{

BWc = pow(BW, CAE); # cardiac scaling output factor (kg)

VplasC = 0.55 * VBldC; # plasma fractional volume

HEME = 1 - (VplasC / VBldC); # Volume of Haeme

VRBC = HEME * VBldC; # Volume of red blood cells

## Gelman reparameterisations

Qcci = QrpdC + QspdC + QhepartC + QfaC + QstC + QguC;

Qrpdci = QrpdC / Qcci;

Qspdci = QspdC / Qcci;

Qhepartci = QhepartC / Qcci;

Qfaci = QfaC / Qcci;

Qstci = QstC / Qcci;

Qguci = QguC / Qcci;

Vti = (1 - VT) + VrpdC + VspdC + VliC +

VfaC + VstC + VguC + VplasC +

VRBC + VlymphC;

Vguci = VguC / Vti;

Vstci = VstC / Vti;

Vfaci = VfaC / Vti;

Vlici = VliC / Vti;

Vspdci = VspdC / Vti;

Vrpdci = VrpdC / Vti;

Vbldci = VBldC / Vti;

Vplasci = VplasC / Vti;

VRBCci = VRBC / Vti;

Vlymphci = VlymphC / Vti;

# Volumes scaled to actual volumes

Vfa = Vfaci * BW; # scaled fractional volume

Vgu = Vguci * BW; # scaled fractional volume

Vst = Vstci * BW; # scaled fractional volume

Vspd = Vspdci * BW; # scaled fractional volume

Vli = Vlici * BW; # scaled fractional volume

Vrpd = Vrpdci * BW; # scaled fractional volume

Vlymph = Vlymphci * BW; # scaled fractional volume

VRB = VRBCci * BW; # scaled red blood cell fractional volume

Vbld = Vbldci * BW; # Whole blood fractional volume

Vplas = Vplasci * BW; # plasma fractional volume

# Calculate actual blood flows from total flow and percent flows

QC = QCC * BWc; # cardiac output (L/h)

Qfa = Qfaci * QC; # scaled fractional blood flow

Qgu = Qguci * QC; # scaled fractional blood flow

Qst = Qstci * QC; # scaled fractional blood flow

Qrpd = Qrpdci * QC; # scaled fractional blood flow

Qspd = Qspdci * QC; # scaled fractional blood flow

Qhepart = Qhepartci * QC; # scaled hepatic artery fractional blood flow

Qli = Qhepart + Qst + Qgu; # scaled fractional blood flow

QCMC = Qhepart + Qgu + Qst + Qfa + Qrpd + Qspd;

} # End of model initialization

Dynamics

{

tau = 8; # the required delay

Ali_lag = CalcDelay(Ali, tau);

AliM_lag = CalcDelay(AliM, tau);

ORALDOSE = PORALDOSE * BW; # scaled oral dose (mg/day)

DOSEFLOW = ORALDOSE / DRINKTIME; # zero order uptake rate constant

ODOSE = DOSEFLOW * DOSESTEP; # amount absorbed (mg)

ODOSEliver = ODOSE * FracDOSEHep;

ODOSElymph = ODOSE * FracDOSELymph;

ODOSEbowel = ODOSE * (1 - FracDOSEHep - FracDOSELymph);

ClintDPHP = (0.693 / DPHP_half_life) * (Incub_vol / Microsome_prot) * MPY * Vli * 60; # Clearance (L/h whole liver)

ClintDPHPgu = (0.693 / DPHP_GUT_half_life) * (Incub_vol / Microsome_prot) * MPYgu * Vgu * 60; # Clearance (L/h gut)

ClintMPHP = (0.693 / MPHP_half_life) * (Incub_vol / Microsome_prot) * MPY * Vli * 60; # Clearance (L/h whole liver)

#********************************************************************************************!

# DPHP Concentrations in Compartments

#********************************************************************************************!

# cellular concentrations (mg/L)

Cfa = Afa / Vfa;

Cgu1 = Agu1 / Vgu;

Cgu2 = Agu2 / Vgu;

Cst = Ast / Vst;

Cspd = Aspd / Vspd;

Crpd = Arpd / Vrpd;

Cli = Ali / Vli;

Clymph = Alymph / Vlymph;

# venous organ concentration (mg/L)

CVfa = Cfa / Pfab;

CVgu1 = Cgu1 / Pgub;

CVgu2 = Cgu2 / Pgub;

CVst = Cst / Pstb;

CVspd = Cspd / Pspdb;

CVli = Cli / Plib;

CVrpd = Crpd / Prpdb;

GPER = KEMAX / (1 + KEMIN * Cst);

# venous concentration (mg/L)

CV =

((CVfa * Qfa) +

(CVrpd * Qrpd) +

(CVspd * Qspd) +

(CVli * Qli)) / QCMC;

# DPHP Venous concentration (nmoles/L)

CVnmol = (CV / MWDPHP) * 1000000;

# Fraction unbound

Aplasmub_DPHP = Aplasm_DPHP * (1 - FB_DPHP);

# mass in system (kg)

mass =

ARBC_DPHP + Aplasm_DPHP + AMli + Ali + ABile + AMgu + ABellyH + AGiTractH +

Ast + Agu1 + Agu2 + ABowel + ABellylymph + AGiTractlymph + Alymph + Afa + Arpd +

Aspd;

Uptake = ODOSEliver + ODOSElymph + ODOSEbowel;

# mass balance

reloral = ((t>0) ? mass / (ORALDOSE + 1e-10) : 1);

CA_DPHP = Aplasmub_DPHP / (Vplas); # Arterial unbound concentration (nmol/L)

#********************************************************************************************!

# MPHP Concentrations in Compartments

#********************************************************************************************!

# cellular concentrations (mg/L)

CguM = AguM / Vgu;

CstM = AstM / Vst;

CfaM = AfaM / Vfa;

CliM = AliM / Vli;

CspdM = AspdM / Vspd;

CrpdM = ArpdM / Vrpd;

# venous organ concentration (mg/L)

CVguM = CguM / PguM;

CVstM = CstM / PstM;

CVfaM = CfaM / PfaM;

CVliM = CliM / PliM;

CVspdM = CspdM / PspdM;

CVrpdM = CrpdM / PrpdM;

CVM =

((CVliM * Qli) +

(CVfaM * Qfa) +

(CVspdM * Qspd) +

(CVrpdM * Qrpd)) / QCMC;

#unbound model

Aplasmub_MPHP = Aplasm_MPHP * (1 - FB_MPHP);

CA_MPHP = Aplasmub_MPHP / (Vplas);

# mass in system (kg)

massMPHP = AguM + AstM + AfaM + AMliM + AspdM + ArpdM + ABowelM + ABileM + ARBC_MPHP + Aplasm_MPHP;

# MPHP mass balance

relMPHP = ((t>0) ? massMPHP / (AMli + AMgu + 1e-10) : 1);

#********************************************************************************************!

# DPHP Differential Equations

#********************************************************************************************!

dt (Gutswitch) = 0;

dt (Lymphswitch) = 0;

dt (DOSESTEP) = 0;

dt (VBladder) = RUrine;

dt (ARBC_DPHP) = (CA_DPHP - CRBC_DPHP / Pbab); # Amount in red blood cells

dt (Aplasm_DPHP) = # Amount in plasma (mg)

QCMC *

(CV - CA_DPHP) -

dt (ARBC_DPHP) +

Lymphswitch * Alymph * K1Lymph;

dt (AMli) = ((Qli * ClintDPHP) / (Qli + ClintDPHP / Pbab)) * CVli; # Amount of hepatic metabolism (mg/h/kg)

dt (Ali) = # Amount in liver (mg)

(Qhepart * CA_DPHP) +

(Qst * CVst) +

(Qgu * CVgu2) -

(Qli * CVli) -

dt (AMli) -

(K1_DPHP_LIVER * Ali);

dt (ABile) = K1_DPHP_LIVER * (Ali - Ali_lag); # Amount in Bile (mg)

dt (AMgu) = ((Qgu * ClintDPHPgu) / (Qgu + ClintDPHPgu / Pbab)) * CVgu1; # Amount of gut metabolism (mg/h/kg)

dt (ABellyH) = (ODOSEliver) - (GPER * ABellyH) - (BELLYPERM * ABellyH); # Amount of in stomach compartment (mg/h/kg)

dt (AGiTractH) = (GPER * ABellyH) - (GIPERM1 * AGiTractH); # Amount of in GI Tract compartment (mg/h/kg)

dt (Ast) = Qst * (CA_DPHP - CVst) + BELLYPERM * ABellyH; # Amount of in STOMACH compartment (mg/h/kg)

dt (Agu1) =

(GIPERM1 * AGiTractH) -

dt (AMgu) -

(Gutswitch * GIPERM2 * Agu1)

;

dt (Agu2) =

Qgu * (CA_DPHP - CVgu2) +

(Gutswitch * GIPERM2 * Agu1) -

#dt (AMgu) -

(Lymphswitch * K1_DPHP_GUT * Agu2) +

(K1_DPHP_LIVER * Ali_lag);

dt (ABowel) = ODOSEbowel + (Lymphswitch * K1_DPHP_GUT * Agu2); # Elimination rate from gut into faeces (mg)

dt (ABellylymph) = # DPHP rate of uptake in lymph compartment (mg/h/kg)

(ODOSElymph) -

(GPER * ABellylymph) -

(BELLYPERMlymph * ABellylymph);

dt (AGiTractlymph) = (GPER * ABellylymph) - (GIPERMlymph * AGiTractlymph); # DPHP rate of uptake in lymph compartment (mg/h/kg)

dt (Alymph) = # Amount in lymph

(BELLYPERMlymph * ABellylymph) +

(GIPERMlymph * AGiTractlymph) -

Lymphswitch * Alymph * K1Lymph;

dt (Afa) = Qfa * (CA_DPHP - CVfa); # cellular compartment derivative (mg/h/kg)

dt (Arpd) = Qrpd * (CA_DPHP - CVrpd); # cellular compartment derivative (mg/h/kg)

dt (Aspd) = Qspd * (CA_DPHP - CVspd); # cellular compartment derivative (mg/h/kg)

#********************************************************************************************!

# MPHP Differential Equations

#********************************************************************************************!

dt (AMliM) = ((Qli * ClintMPHP) / (Qli + ClintMPHP / PbaM)) * CVliM;

dt (AliM) =

(Qhepart * CA_MPHP) +

(Qst * CVstM) +

(Qgu * CVguM) -

(Qli * CVliM) +

dt (AMli) -

dt (AMliM) -

(K1_MPHP_LIVER * AliM);

dt (ABileM) = K1_MPHP_LIVER * (AliM - AliM_lag);

dt (AstM) = Qst * (CA_MPHP - CVstM);

dt (AguM) =

Qgu * (CA_MPHP - CVguM) +

dt (AMgu) -

(Lymphswitch * K1_MPHP_GUT * AguM) +

(K1_MPHP_LIVER * AliM_lag);

dt (ABowelM) = K1_MPHP_GUT * AguM;

dt (AfaM) = Qfa * (CA_MPHP - CVfaM);

dt (AspdM) = Qspd * (CA_MPHP - CVspdM);

dt (ArpdM) = Qrpd * (CA_MPHP - CVrpdM);

#unbound model

dt (ARBC_MPHP) = (CA_MPHP - CRBC_MPHP / PbaM);

dt (Aplasm_MPHP) = QCMC * (CVM - CA_MPHP) - dt (ARBC_MPHP);

#********************************************************************************************!

# MPHP Urinary excretion

#********************************************************************************************!

dt (AMMPHPB_MOH) =

dt (AMliM) * FracMetabMOH * (MWMPHPOH / MWMPHP) -

(K1_MOH * AMMPHPB_MOH);

dt (AMMPHPB_cx) =

dt (AMliM) * FracMetabcx * (MWMPHPcx / MWMPHP) -

(K1_cx * AMMPHPB_cx);

dt (AMMPHPU_MOH) = K1_MOH * AMMPHPB_MOH;

dt (AMMPHPU_cx) = K1_cx * AMMPHPB_cx;

Curine_MOH = K1_MOH * AMMPHPB_MOH;

Curine_cx = K1_cx * AMMPHPB_cx;

}

#These are new addition. Also specified in final four lines of 'outputs'

CalcOutputs

{

CAT_DPHP = Aplasm_DPHP/(MWDPHP*Vplas)*1e6; # Total concentration in plasma (nmol/l)

CV_total_nmol = (Aplasm_DPHP+ARBC_DPHP)/(MWDPHP*Vbld)*1e6; # Total concentration in blood (nmol/l)

CRBC_DPHP = ARBC_DPHP / (MWDPHP*VRB)*1e6; # Concentration in red blood cells (nmol/L)

CAT_MPHP = Aplasm_MPHP / (MWMPHP*Vplas)*1e6;

CRBC_MPHP = ARBC_MPHP / (MWMPHP*VRB)*1e6;

CV_total_MPHP_nmol = (Aplasm_MPHP+ARBC_MPHP)/(MWMPHP*Vbld)*1e6;

Plasma_DPHP = (Aplasm_DPHP + ARBC_DPHP)/(Vbld);

Plasma_MPHP = (Aplasm_MPHP + ARBC_MPHP)/(Vbld);

Urine_cx = AMMPHPB_cx*K1_cx;

Urine_OH = AMMPHPB_MOH*K1_MOH;

}

End.
